# Epidermal Growth Factor Intralesional Delivery in Chronic Wounds: The Pioneer and Standalone Technique for Reversing Wound Chronicity and Promoting Sustainable Healing †

**DOI:** 10.3390/ijms252010883

**Published:** 2024-10-10

**Authors:** Jorge Berlanga-Acosta, Ariana Garcia-Ojalvo, Jose Fernández-Montequin, Viviana Falcon-Cama, Nelson Acosta-Rivero, Gerardo Guillen-Nieto, Merardo Pujol-Ferrer, Miladys Limonta-Fernandez, Marta Ayala-Avila, Elof Eriksson

**Affiliations:** 1 Wound Healing, Tissue Repair and Cytoprotection Research Project, Biomedical Research Direction, Center for Genetic Engineering and Biotechnology, Playa, P.O. Box 6162, Havana 11600, Cuba; ariana.garcia@cigb.edu.cu (A.G.-O.); vivianyart@gmail.com (V.F.-C.); nelson.acosta@fbio.uh.cu (N.A.-R.); gerardo.guillen@cigb.edu.cu (G.G.-N.); merardo.pujol@cigb.edu.cu (M.P.-F.); miladys.limonta@cigb.edu.cu (M.L.-F.); marta.ayala@cigb.edu.cu (M.A.-A.); 2 National Institute of Angiology and Vascular Surgery—Diabetic Angiopathy Service, Calzada del Cerro 1551 esq, Domínguez, Cerro, Havana 12000, Cuba; montequi@infomed.sld.cu; 3 Joseph E. Murray Professor of Plastic and Reconstructive Surgery, Brigham and Women’s Hospital, Harvard Medical School, Main Pike, ASB-2, 75 Francis St, Boston, MA 02115, USA; elof@comcast.net

**Keywords:** EGF, diabetic foot ulcers, growth factors, chronic wounds, ulcers, wound healing

## Abstract

The early expectations about growth factors’ (GFs’) discovery as an undisputed therapeutic solution for chronic wounds progressively eclipsed when they failed to accelerate acute wound closure and restore the healing trajectory of stagnant ulcers. Critical knowledge about chronic wound biology and GF pharmacology was a conundrum at that time. Diabetes undermines keratinocytes’ and fibroblasts’ physiology, impairing skin healing abilities. Diabetic ulcers, as other chronic wounds, are characterized by hyperinflammation, unbalanced proteolytic activity, catabolism, and free radical cytotoxicity. This hostile scenario for the chemical stability, integrity, and functionality of GFs led to the conclusion that topical administration may jeopardize GFs’ clinical effectiveness. Epidermal growth factor (EGF) has a proximal position in tissues homeostasis by activating survival and mitogenic pathways from embryonic life to adulthood. Seminal experiments disclosed unprecedented pharmacological bounties of parenterally administered EGF. Accordingly, the experience accumulated for more than 20 years of EGF intralesional infiltration of diabetic wound bottoms and edges has translated into sustained healing responses, such as low recurrences and amputation rates. This delivery route, in addition to being safe and tolerated, has shown to restore a variety of circulating biochemical markers ordinarily disturbed in diabetic conditions. EGF infiltration triggers a cascade of local fibroblast reactions, supporting its molecular integrity, prolonged mean residence time, and ultimately eliciting its receptor trafficking and nuclear translocation. The intralesional delivery route seems to warrant that EGF reaches wound fibroblasts’ epigenetic core, mitigating the consequences of metabolic memory imprinting.

## 1. Significance

1. This review is an analytical description of the history of growth factors, particularly of epidermal growth factor (EGF), as the inaugurator of a new era in which this and other polypeptides were expected to enhance the closure of acute wounds and reverse the chronicity phenotype of stagnant wounds as such as diabetic, venous, and pressure ulcers. The biological bases for this rationale in the early stages appeared to be well founded. However, after more than 40 years of research GFs have not conquered a definitive pharmacological niche. Likewise, the FDA has not granted clearance for a GF-based medication for wound healing since 1997.
2. The introduction of GFs into the clinical scenario appeared to be premature when critical aspects of both the molecular basis of wound chronicity and GF pharmacology had not been identified. Seminal pieces of the GFs’ local pharmacokinetics in their interaction within the wound microenvironment were found later, concluding that wounds with a pathologic inflammatory state and a high proteolytic microenvironment are a hostile environment for GFs, their receptors, and, consequently, to their biological activity.
3. Here, we describe our experience with an unprecedented interventional delivery route which has conclusively leveraged EGFs’ intrinsic biological capabilities. This is based on the intra- and perilesional infiltration of EGFs directly into the wound bed and dermo-epidermal junction of the epithelial edges of diabetic complex, high-grade, neuropathic and ischemic ulcers and stagnant amputation residual wounds. Twenty years of medical practice has shown that beyond the complex biochemistry of a chronic diabetic wound, the appropriate delivery of a GF may restore the “acute healing phenotype” and vindicate what the lonesome EGF can do for reversing the chronicity program.

## 2. Introduction

The germane studies by Viktor Hamburger during the 1940s were transcendental for developmental biology and medicine given his timely presumption of the existence of inductive, organizer agents for cell differentiation, growth, and survival. His ideas sowed the seeds for the restless search for what we know today as growth factors (GFs) [1]: the ultimate temporal–spatial organizers responsible for embryonic pattering, organogenesis, and development [2].

The subsequent encounter between Rita Levi-Montalcini and Stanley Cohen years later steered researchers towards the identification of a protein with nerve growth activity: the nerve growth factor (NGF). Cohen, having observed the non-neuronal effects of the crude extract of salivary glands on newborn mice, identified the second GF as epidermal growth factor (EGF). It was originally named “tooth eyelid factor”. For their discovery of NGFs and EGFs, Levi-Montalcini and Cohen shared the 1986 Nobel Prize [3].

GFs are a family of polypeptides that imprint cells’ phenotypic destiny, location, and behavior through binding to specific cell surface receptors, most of them endowed with tyrosine-kinase activity, subsequently activating conserved signaling pathways [4]. Given that GFs are significant drivers of cell growth, proliferation, migration, and differentiation, their guidance begins during the preimplantation stages of embryonic life [5,6]. In post-natal life, GFs are ubiquitous ingredients in most body fluids and epithelial and mesenchymal tissues of mammals, not only guiding healing events, but ensuring epithelial cell populations’ homeostatic turnover [7]. Moreover, a large body of evidence illustrates the variegated role of GFs in malignant cells’ transformation events, including metabolic reprogramming, multidrug resistance, self-sufficiency, and remote metastatic seeding [8,9,10]. The early concept of external GFs’ independence supply due to autocrine and paracrine loops for malignant cell survival shed light on the ability of GFs to trigger unexpected cell-adaptive and plasticity capabilities before otherwise lethal insults [11]. In other words, since those early days, a wide horizon of GFs’ biological properties were progressively described and associated with regenerative medicine [12,13].

The early beneficial effects of exogenous GFs in the treatment of wounds, as well as in the identification of the in vitro activities of many GFs, implicated these proteins as key regulators in the wound healing process [14]. This notion has been historically supported by the expression of multiple GFs and their receptors in different cell types during skin and other tissue/organ repairs [15,16,17,18].

All organisms from single-cell amoebae through to *Homo sapiens* have evolved strategies for repairing wounds as an essential homeostatic mechanism of reconstructing damaged structures [19]. The healing process is a complex sequence of overlapping stages supported by essential biological functions such as cellular proliferation, migration, secretion, differentiation, and transdifferentiation. The process is subdivided into (i) hemostasis, (ii) inflammation, (iii) proliferative/granulation, and (iv) remodeling [20]. The quality, accuracy, and precision in the cascade of healing events is safeguarded by GF signals, primarily released from platelet alpha granules during the hemostasis phase [21,22]. Thus, it is understood that each GF or family of GFs is endowed with temporary responsibilities according to the wound’s transitional phase [23]. Different GF families have been studied within the healing process, such as the following of major significance: EGF, PDGF, FGF, IGF, VEGF, and the pro-fibrogenic TGF-β. Accordingly, a sort of roadmap of GF physiology in normal wound healing is beginning to be devised today [24]. Likewise, seminal studies indicate that some GFs, like EGFs, appear to be physiologically irreplaceable, given their proximal position within the cascade of tissue protection and repair signalers [25,26,27,28]. Conversely, evidence indicates that GF deficits are linked to the orchestration of wound chronicity phenotype. Chronic wounds, characterized by their pathologic inflammatory state and proteolytic microenvironment, are a hostile substrate for GFs’ and their receptors’ integrity and function [29]. In some types of chronic wounds, the deficit in GFs may appear as a primary trigger [30,31,32]. It has therefore been proposed that reductions in GF availability are a priming factor for the onset and perpetuation of a senescent cell community as a pillar of chronicity [33].

The history of GF pharmacology in wound healing armamentarium has not historically progressed along a smooth road. The first clinical intervention with a recombinant human GF dates back to 1989, when Brown and co-workers topically administered EGFs to accelerate the regeneration of skin graft donor sites in burn victims [34]. However, despite years of praised success upon the topical administration of GFs, it is perplexing that Regranex remains the only GF-based medication approved (1997) by the FDA for the treatment of diabetic foot ulcers (DFUs). The therapeutic success of Regranex has been questioned even when it is indicated for low-grade ulcers with good prognosis and is based on PDGF-BB, a deficitary GF in diabetic ulcers called to act as a replacement therapy [35]. We deem that the clinical use of GFs was somewhat premature. Critical aspects of both the molecular basis of wound chronicity and GF pharmacology had not been identified at the time. On top of that, most of the experimental wound models used to examine the capabilities of exogenously applied GFs are far from mirrored human pathology [36]. Notably, these adversities were all derived from topically administered GFs, which reinforces the notion that GFs are not suitable candidates for topical administration due to their reduced pharmacodynamics [37]. These results eclipsed the hyper-enthusiasm of early years when GFs were envisioned as “magic healing bullets”. Today, a revolution of novel and complex delivery systems and nanoformulation strategies is escalating at the preclinical level, which may ensure an appropriate local residence time, penetrability, and, accordingly, biological activity. They all, however, will need years to prove their utility in clinical trials [13,38,39,40].

This review describes our experience with an unprecedented interventional delivery route which has conclusively leveraged EGFs’ intrinsic biological capabilities. This is based on the intra- and perilesional infiltration of EGFs directly into the wound bed and dermo-epidermal junction of the epithelial edges of diabetic complex, high-grade, neuropathic and ischemic ulcers and stagnant amputation residual wounds. The rationale for this delivery stems from basic findings that justified the route as a channel for the intra-tissue delivery of a stable, integral molecule, ensuring an appropriate residence time, allocating EGFs in deep layers populated by sensitive fibroblasts, and ultimately transforming the ancestral paradigm of superficial wound treatment. Twenty years of medical practice has shown that beyond the complex biochemistry of a chronic diabetic wound, the appropriate delivery of a GF may restore the “acute healing phenotype” and vindicate what the lonesome EGF can do to reverse the chronicity program. Moreover, this is an adjunctive pharmacological intervention that is incorporated into the medical armamentarium and does not replace any of the classical standard-of-care tools.

## 3. Brief Overview of the EGF System Biology

**Figure 1:**
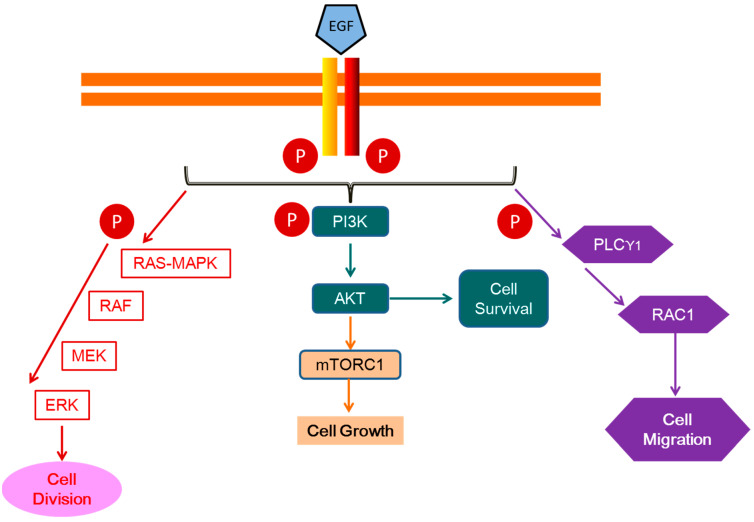
Simplified schematic representation of EGFR activation and pathways. EGFR is rapidly activated via autophosphorylation when a ligand like EGF is bound. This extracellular mitogenic signal activates a cascade of downstream signaling pathways including MAPK-Ras/ERK, which ultimately promotes cell proliferation. Phosphorylation of the PI3K/Akt pathway triggers cell survival through the activation of anti-apoptotic mechanisms. Alternatively, AKT can also activate the mTORC1 complex, which enhances cellular synthesis of lipids and proteins. Activation of PLCγ1 the pathway is associated with cytoskeleton organization and cell motility. EGF: epidermal growth factor; MAPK: mitogen-activated protein kinase; ERK: extracellular-regulated kinase; PI3K: phosphatidylinositol 3-kinase; mTORC1: mammalian target of rapamycin complex 1; PLCγ1: phospholipase C gamma 1.

Although a description of the EGFR signaling pathway is beyond the scope of this review, we deem appropriate to remind readers of the functional aspects of the EGF/EGFR system (Figure 1) as an emblematic tyrosine-kinase activity receptor.

EGF is perhaps the most largely and deeply studied GF because it seems to be necessary and sufficient to orchestrate elemental mechanisms for cellular viability such as survival, anabolism, and proliferation [41]. As a matter of fact, EGF’s first functional clue was revealed by its ability to accelerate internal pacemakers in mice, regulating the post-natal maturation of epithelial appendages, which we interpret as the temporary reprogramming of evolutionarily imprinted events [25]. Subsequent studies have demonstrated the competence of exogenously administered EGF to accelerate the functional reprogramming of intestinal epithelial cells upon stressful scenarios [42,43]. Both events are likely supported by EGF’s ability to modify the epigenetic program of epithelial cells.

EGF is accompanied in its family by TGF-α, heparin-binding EGF-like growth factor (HB-EGF), amphiregulin, epiregulin, betacellulin, neuregulins, and epigen. These ligands exert their functions by selective binding to four different high-affinity receptors, EGFR/ErbB1, HER2/ErbB2, HER3/ErbB3, and HER4/ErbB4. EGFR/ErbB1, which is occupied by EGF, has an ample organismal distribution, being expressed by almost all cell types with the exception of hematopoietic cells [44]. This family of receptors can form a total of 28 different combinations with each other, which contributes to the robustness of the signal upon selective sensing of “who the ligand is” [45]. Thus, the activation of the EGFR by different ligands has been shown to cause distinct downstream biological activities [46]. Upon its stimulation, EGFR is internalized, trafficked through different compartments, recycled, and also translocated to the nucleus, where it plays an active role in cell proliferation and DNA repair [47]. One major pathway downstream of EGFR and other tyrosine-kinase receptors is the mitogen-activated protein kinase (RAS-MAPK) pathway, which is involved in cell proliferation. In a similar manner, EGFR family members recruit phosphatidylinositol 3-kinase (PI3K), whose product PIP3 attracts AKT kinase, which is ultimately activated by both PDK1 and mTORC2 (Figure 1). After its activation, AKT prevents apoptotic cell death by phosphorylating BAD and FOXO transcription factors. The activation of the mTORC1 complex promotes protein and cholesterol biosynthesis. PLCγ1 is another phosphorylation target of activated EGFR and is committed in cell motility/migration [48]. The addition of EGF to an epithelial cell induces the expression of its own receptor by stimulating the expression of EGFR-specific transcription factor (ETF) [49]. More recent studies have shown that EGFR, activated upon EGF binding, causes phosphorylation of 2244 proteins at 6600 sites and a differential expression in 3172 genes in human mammary epithelial cells (HMECs) [50]. Nevertheless, and in a summarized manner, EGF is implicated in the control of cellular metabolism, proliferation, cell size, survival, and motility [51,52] (Figure 1).

## 4. EGF: Biological Virtues and Pharmacological Bounties

Here, we describe the biological activities and pharmacological bounties that may be induced by EGF, as an endogenous ligand of HER-1, or upon its exogenous administration at pharmacological concentrations. Accordingly, different lines of evidence document the relevance of the EGF family of ligands and their receptors for skin and appendage physiology and homeostasis. The dysregulation of this system may result in impaired wound healing [53,54,55], with diabetes as the main exemplary scenario [56]. Classic evidence from more than 30 years ago congruently documents that (I) the EGF/EGFR-mediated pathway is activated during wound repair, (II) EGF and its receptor are expressed by skin keratinocytes, fibroblasts, and vascular endothelial cells, which are all involved in skin repair, (III) EGF administration enhances the epidermal regeneration of partial thickness burns, (IV) the wound tensile strength of skin incisions increases in rats treated with EGF, (V) wounding induces immediate shedding of EGFR ligands from keratinocyte membranes, facilitating re-epithelialization, and (VI) EGF acts as a survival factor for fibroblasts in a concentration-dependent manner. In line with this, (VII) EGF treatment enhances procollagen mRNA expression due to increased fibroblast proliferation, whereas (VIII) it also increases wound contraction by activating myofibroblast proliferation and collagen deposition. (IX) EGF regulates the production of free radicals by keratinocytes, which stimulates cell proliferation and inflammation resolution following wounding; accordingly, (X) EGF enhances the expression of antioxidant defense enzymes by accelerating gene transcription, resulting in cell protection from the effects of superoxide radicals [57,58,59,60,61,62,63,64,65]. More recent studies document that EGF, but not other GFs, induces the development of front–rear polarity and directional migration of keratinocytes, which is relevant for re-epithelialization [66]. Another distinctive feature of EGF, but of no other GFs, is its ability to downregulate and counteract inflammation [67]. Primary cultures of dermal fibroblasts survived when incubated with EGF before the cytotoxic effects of free radicals and pro-inflammatory cytokines [68]. Other data have also described its anti-inflammatory and anti-pigmentation effects [69]. EGF has also been shown to induce hair follicle mesenchymal stem cell proliferation through the EGFR/ERK and AKT pathways with a concomitant inhibition of p16 expression, suggesting a pro-rejuvenation effect in skin cells [70]. The potential rejuvenating or senolytic effects of EGF may be a unique attribute of this GF. Aged fibroblasts exhibit improved migratory and contractile responses when cultured with EGF compared with young donor-derived fibroblasts cultured under 3D conditions [71]. A groundbreaking study revealed that cell cultures depleted of EGF progress toward a senescent phenotype with decreased proliferation, reduced Rb phosphorylation, and elevated p21 expression. Therefore, EGF dependence may be a mechanism to escape from senescence and ensure proliferation, putatively placing EGF as a central primer for mitogenic competence and senescence prevention [72,73]. Likewise, EGF has been shown to activate the expression of human telomerase reverse transcriptase in cultured cells via Ets-2, a transcription factor that appears to depend on EGFR activation [74]. An increasing line of evidence promotes that EGF prevents and reverses skin cell injuries resulting from ionizing radiation, which confirms the unique cytoprotective input of this GF [75,76,77]. Recent studies attempting to characterize cells’ metabolic phenotypes during the healing process have highlighted the significance of the metabolic programming of glucose, lipids, and amino acids for successful transit through the healing phases [78]. It has recently been demonstrated that EGF stimulates wound cells’ metabolic reprogramming from oxidative phosphorylation to aerobic glycolysis by the upregulation of glycolytic enzymes and glucose uptake transporters [79,80]. Since broad metabolic reprogramming events are conducted by Myc upon EGFR agonistic activation [81], it may be tenable to suggest that EGF administration may contribute to wound cells’ reprogramming beyond aerobic glycolysis [82,83]. Conclusively, wound cell metabolic reprogramming is a newly identified EGF biological bounty, and may represent an additional checkpoint for which this GF guides the healing process.

## 5. Drivers Supporting EGF Intralesional Infiltration

Diabetes is associated with a particular deficit of circulating [84] and salivary levels of EGF [85], which synergizes with the glucotoxic environment to drive multicellular system demise [86]. Hence, exogenous administration of the GF may act as a replacement therapy for wound cells’ biological competence [87]. The experiments in the early 1990s, in which we examined the response of locally infiltrated EGF to injured sciatic nerves in rats, marked a significant milestone for EGF’s definitive drugability as an injectable medication. It contributed to reprofiling the molecule from its traditional topical route. These pioneering studies sought to understand the neurological response to EGF locally administered in rats with hind limb denervation by sciatic nerve transection. The animal model somewhat mirrored a diabetic-like combination of neuropathy and tissue hypoxia. In situ EGF infiltration not only restored remyelination, but also improved the survival of soft tissues in the hind limbs and reduced the occurrence of plantar ulcers and toe necrosis [88]. We subsequently showed, in a variety of pathological models, that single or repeated EGF systemic injections or local infiltrations stimulated “clear-cut” cytoprotective and proliferative responses, supporting the intrinsic ability of EGF at supraphysiological concentrations to promote tissue repair [25,89]. The idea of EGF parenteral administration was further encouraged when it came to light that EGFR is located only on the basolateral, but not in the apical surfaces of epithelial cells, thus suggesting that blood-transported, but not apical EGF, was the one present in epithelial cell physiology [90,91].

**Figure 2:**
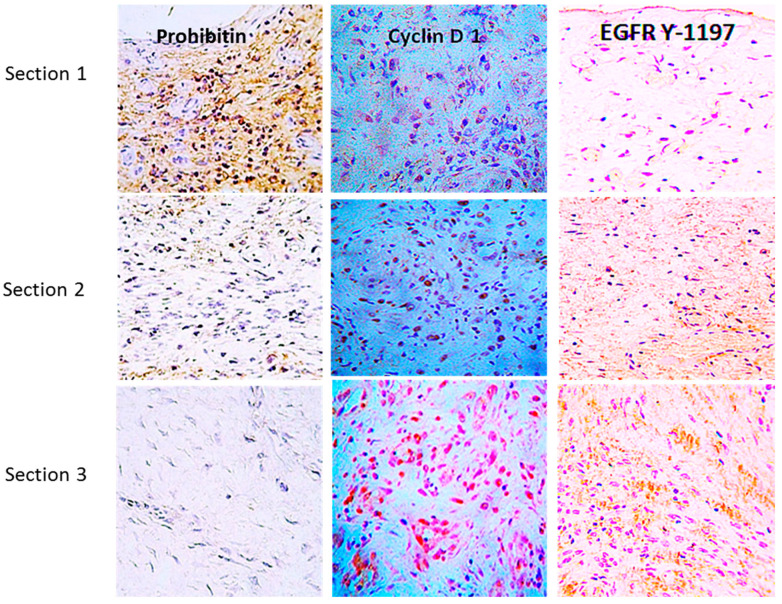
Immunohistochemical characterization of proliferation-related markers. Three sections (≈2–3 mm length) are immunohistochemically distinguished in biopsy materials by immunolabeling with antibodies against prohibitin as a cell-cycle negative regulator and cyclin D1 as a master switch controlling G1–S transition in response to growth factor stimulation. The active EGFR phosphorylated in tyrosine 1197, as a critical substrate for multiple physiological functions of the receptor, is shown. Prohibitin is far more abundant in the most superficial layer of the wound. Conversely, cyclin D1 and EGFR Y-1197 appear marginally labeled. Cyclin D1 and the phosphorylated form of EGFR are abundantly detected in Section 3.

Injecting EGF down into the base and contours of the wounds, including the derma-epidermal junction, contributed to the following: (I) the direct delivery of the GF to the responsive cells, (II) reductions in its local degradation, (III) jumping over the diffusion-limiting barriers from the wound surface to the deeper stratum, and (IV) ensuring EGF bioavailability for a prolonged interaction with the receptor in deep layer cells. The immunohistochemical demonstration of the existence of a cellular distribution of EGFR and other cell proliferation regulators along the longitudinal axis of ulcer tissue beds was a crucial hint for the rationale of the local infiltration treatment. In parallel, the finding contributed to explaining why topical administration of GFs had failed. As shown in Figure 2, EGFR is not detected on the wound surface’s cell layer, in contrast to deeper cells’ strata, where it is intensely expressed.

**Figure 3:**
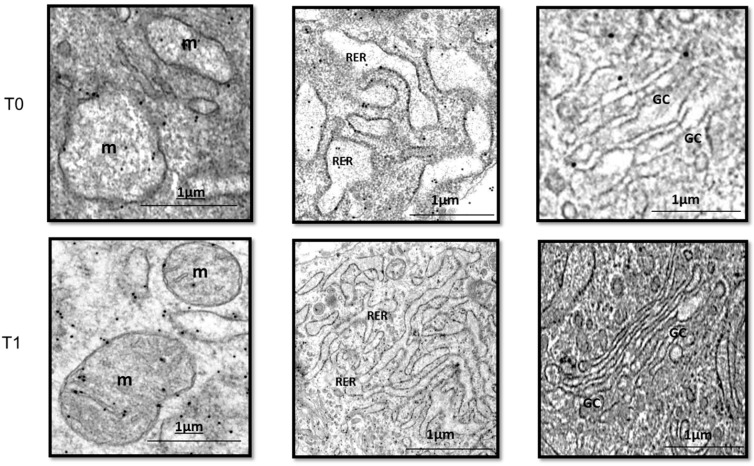
EGF-mediated ultrastructural preservation of ulcer fibroblast organelles. Upper panel. Organelles of a fibroblast from a diabetic ulcer biopsy before EGF infiltration (T0). Left image—m: mitochondria exhibiting dilation and total cristae lysis. Central image—RER: rough endoplasmic reticulum exhibiting cisternae system dilation. Right image—GC: Golgi complex, showing dilation and fragmentation of the tubular system. Lower panel. Representation of the morphological transformation of organelles of fibroblasts 24 h after EGF infiltration (T1). Mitochondria appear less dilated and with incipient cristae. RER and GC exhibit normal morphology. Bar = 1 μm.

**Figure 4:**
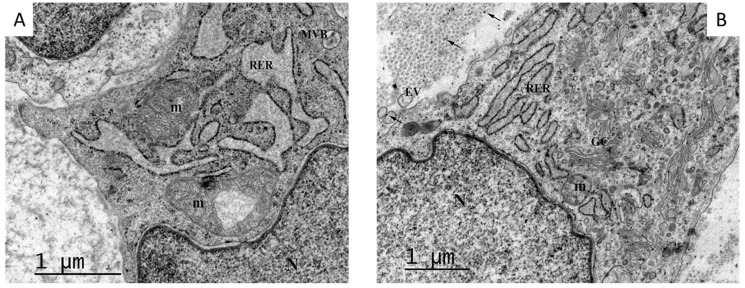
EGF-mediated EGFR activation and polarization. (**A**) Fibroblast cell from biopsy of a diabetic foot ulcer sample obtained before EGF infiltration. Negligible EGFR expression and scarce extracellular vesicles are observed. (**B**) Fibroblast sample derived from an ulcer biopsy 24 h after local infiltration of EGF. EGFR labeling indicates its encapsulation in intraluminal membranes of multivesicular bodies (MVBs)—indicated by arrows—and in extracellular vesicles (EVs) adjacent to plasma membrane. m: mitochondria; RER: rough endoplasmic reticulum. (Bar = 1 μm).

Prohibitin, a renowned tumor suppressor and cell cycle arrest protein [92], is far more abundantly expressed in the upper section corresponding to the most superficial stratum of the wound. Conversely, cyclin D1 and EGFR Y-1197 appear marginally labeled. It is noticeable that proliferative cyclin D1 and the phosphorylated form of the EGFR are abundantly detected in the bottom section—the deepest wound layer where no prohibitin is detected. Similarly, cyclin D1, the G1-S phase cell cycle promoter [93], is not expressed by wound superficial cells but is in lower cell layers. Other early studies by our group showed that ^125^I-EGF formulated in a semisolid vehicle appeared to be rapidly cleared from the application site, probably by protease-driven cleavage and receptor-mediated endocytosis. Mean residence time values suggested that over 60% of the administered amount may have disappeared as early as two hours post-administration [94]. This local pharmacokinetic modeling based on topical EGF administration indicated that the receptors dynamics do not fulfill the requisite of prolonged receptor/ligand interaction for wound cell proliferation [95]. Our reasoning was further supported by the evidence provided by Cross and Roberts in 1999, showing a limited kinetic absorption and meager penetration of GFs to deep tissue layers [96]. Another experiment by our group concluded that the clean exudate of acute, controlled, full-thickness wounds in pigs, under laboratory conditions, contained proteolytic activity that may also jeopardize EGF integrity and stability [97]. The most robust experimental support of our intralesional delivery approach, derived from a time-point immunoelectron microscopy kinetic study, in which we visualized the intracellular trafficking of the EGFR in ulcer fibroblasts after EGF infiltration, in a clinical setting [98]. The study showed that locally infiltrated EGF into Wagner’s III and IV neuropathic ulcers resulted in (a) acute morphological restoration of different fibroblasts–intracellular organelles (Figure 3); (b) an increase in EGFR expression 15 min after EGF infiltration as compared to the pre-infiltration point, which indicated the induction of the receptor by the ligand; (c) immediate endocytosis of the EGFR/ligand complex; (d) translocation and biodistribution to different cytoplasmic organelles from 15 min to 24 h after the infiltration; (e) nuclear translocation of EGFR and its binding to DNA, which appeared to last up to 24 h after the treatment; (f) a concomitant activation of the proliferating cell nuclear antigen (PCNA) gene transcription 24 h after the injection; and (g) significant and intriguing accumulation of the receptor in the mitochondria, which lasted for 24 h after the infiltration. Importantly, this work also revealed the polarized accumulation of EGFR-containing exosome-like extracellular vesicles in fibroblast membranes (Figure 4) [98], aligning with previous descriptions of polarized basolateral clusters of EGFR in human keratinocytes exposed to immobilized EGF and indicating enhanced signal transduction [48]. Collectively, these findings endorse the EGF intralesional infiltrative delivery procedure as an effective method to overcome the historical challenges of topical GF administration.

## 6. EGF as the Standalone GF in Healing High-Risk Diabetic Wounds

**Figure 5:**
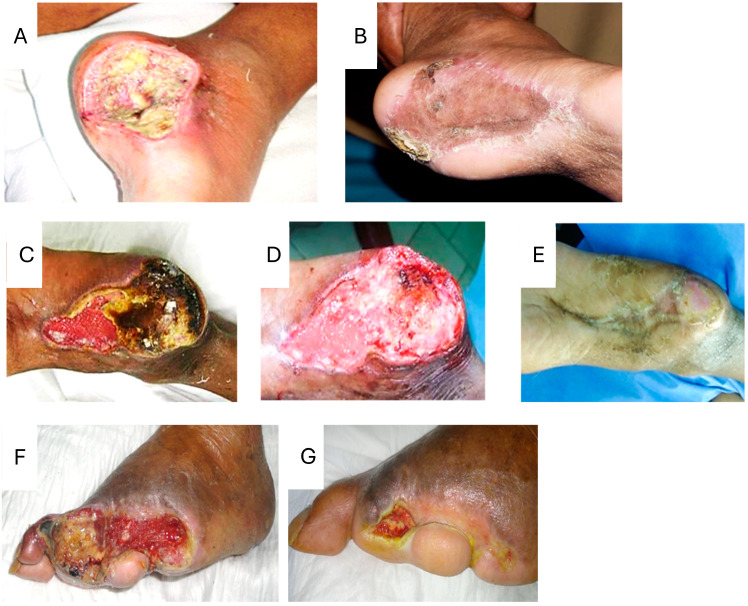
Graphic evidence of the type of diabetic lower limb wounds treated with locally infiltrated EGF. (**A**) Calcaneal lesion at time 0 prior to EGF infiltration. (**B**) shows a free skin graft take which was practiced 8 weeks after EGF intervention, when abundant and productive granulation tissue replenished the wound area. (**C**) shows the aspect of another calcaneal lesion with an ischemic plaque at time 0. (**D**) shows how six weeks of treatment with EGF generated a productive granulation tissue and allowed for the detachment of the ischemic plaque. (**E**) shows how spontaneous re-epithelialization began on week 7 and progressed until complete closure on week 11. EGF infiltration sessions concluded on week 8. (**F**) shows time 0 pre-treatment. EGF infiltration was initiated three weeks after the wound became arrested and no further granulation tissue was produced. (**G**) shows how the lesion resumed granulation and began to contract upon EGF treatment. The image is representative of 7 weeks of treatment.

Compelling evidence from about 10 years of experimental pharmacology in a variety of pathologic models, doses, and administration regimens prompted the need for the development of an injectable pharmaceutical composition for EGF. Pre-formulation and stability tests demonstrated that the mixture of sucrose and dextran resulted in unsurpassed chemical, physical, conformational, and biological stability rates [99]. The first clinical study began in 2001 including 29 diabetic subjects with high-grade (Wagner scale III and IV), poor-prognostic, ischemic and neuropathic refractory-to-heal ulcers and post-amputation residual bases. EGF was infiltrated within the bottom and edges of the lesions thrice per week, initiating in areas of exposed periosteum, cartilage, fascia, and tendon to be covered with granulation tissue in minimal time. In this trial, 17 patients who completed 5 to 8 weeks of treatment re-epithelialized their wounds and showed no recurrence upon a 12-month follow-up. Biopsies collected prior to and during 9 to 12 EGF infiltrations demonstrated that the GF intervention improved the histological aspect of neuropathic and ischemic lesions by increasing wound matrix productive cellularity with secreting fibroblasts, enhancing collagen deposition and increasing the amount of active blood vessels with a concomitant reduction in the inflammatory infiltrate [100]. These findings justified the organization of a clinical trial program to fully discern if the medication/intervention could become an adjunctive treatment to the conventional standard of care of this pathology. A multicenter, double-blind trial that randomized between 25 µg and 75 µg dose levels in each application was implemented. Similarly, it involved infiltrations three times/week until complete granulation or 8 weeks in 41 ischemic and neuropathic patients with Wagner scale III and IV lesions. Complete granulation was obtained in 82.6% and 61.1% of patients at 3.8 and 4.9 weeks of treatment as a median in the 75 µg and 25 µg groups, respectively. Complete epithelialization at 20 weeks after initiation of treatment occurred in 56% and 50% of patients, respectively [101]. A confirmatory, multicenter, randomized, placebo-controlled and double-blind study was carried out, where three treatment groups were compared (EGF 75 µg, 25 µg, and placebo) in patients with advanced diabetic foot ulcers, more than 50% of which were ischemic and with more than 20 cm^2^ as the median initial area of the lesions. At the end of treatment, the proportion of patients with a complete granulation response was significantly higher for the 75 µg dose (87%) compared to the placebo (58%). The time to complete granulation tissue coverage was 3 weeks in both groups receiving EGF and 5 weeks in the placebo group. The 12-month follow-up showed that recurrence rates were low in those subjects treated with EGF 75 µg [102]. Two independent and extemporaneous pharmacovigilance studies [103,104] indicated that intralesional EGF infiltration in chronic lesions with diabetic subject results in a 75% full granulation response, 61% wound closure, and a 71% reduction in amputation-relative risk, as well as a positive benefit–risk balance. With Figure 5, we illustrate representative wounds treated with local EGF infiltration. All were refractory to heal for about 2 to 3 weeks, classified as Wagner grades III and IV, and anatomically/topographically classified as complex wounds.

Of major importance is that survival was longer in patients who had achieved ulcer healing than in those who did not heal [103]. It is also worth noting that with this intervention, recurrence did not exceed 5% upon a 12-month follow-up. Recurrence after the primary re-epithelialization is a serious unresolved medical challenge. A classic study reveals that roughly 40% of diabetic patients have a recurrence within one year after ulcer healing [105]. The recent analysis of 24 published clinical trials evaluating cutting edge treatments, such as Regranex, Apligraf, Dermagraft, Epifix, and vacuum-assisted closure (VAC), showed that recurrence rates were reported in only 10 studies (41.6%). EGF treatment based on intralesional infiltration delivery appeared to provide the longest remission times and the best healing responses [106,107]. It is likely that this remission-prevention outcome by infiltrated EGF may be related to a local “reprogramming effect” on scar-resident fibroblasts’ epigenetic memory [106]. Moreover, the treatment also contributes to improve different diabetes-related biochemical circulating markers. Nine to twelve EGF infiltrations significantly reduced circulating levels of oxidative stress parameters (total oxidative capacity, advanced oxidation protein products, malondialdehyde, and total organoperoxides) and increased systemic antioxidant reserve markers (total antioxidant capacity and sulfhydryl groups). At least half of the patients responded positively to EGF infiltration in terms of redox balance markers, which was associated with a good clinical response in terms of granulation, contraction, and re-epithelialization [108]. A second study [109] not only corroborated the systemic antioxidative effect of intralesional EGF infiltrations, but also showed that this therapeutic approach is capable of attenuating the inflammatory state associated with patients with DFU. The erythrocyte sedimentation rate, as well as circulating levels of IL-6, soluble FAS, MIP1-α, and pentosidine were significantly diminished after nine EGF infiltrations (Table 1). Systemic nitrosilative stress parameters were also reduced. Finally, the introduction of this EGF intralesional infiltration in the daily practice of international groups has confirmed the high healing responses associated with low amputation and recurrence rates [110,111,112]. We deem that EGF/EGFR axis activation acts like a proximal master switch in the healing process through direct downstream signaling or trans-regulatory pathways, which ultimately coordinates and drives a cascade of molecular events spanning from fibroblast metabolic reprograming to keratinocyte polarity and migration.

## 7. Concluding Remarks

Chronic wounds remain a worldwide social and medical problem associated with high morbidity and mortality. Despite extensive scientific efforts and financial investments, diabetic limb wounds and venous and decubitus/pressure ulcers still demand more effective therapeutic strategies. The discovery and introduction of GFs in the clinical arena aimed to overcome these wounds’ chronicity phenotype and restore an acute healing program. Those early days’ expectations progressively faded away when chronic ulcers appeared refractory to heal upon topical GF administration. Chronic wounds are a group of cutaneous injuries with a particular environmental biochemistry and self-interconnected circuits that perpetuate the “open portal phenotype”. When the clinical use of GFs was inaugurated, several aspects of chronic wound biochemistry were a conundrum. Similarly, critical pieces about GFs’ biochemistry puzzle were also missing. Most importantly, seminal aspects of the GFs’ pharmacokinetics in their interaction within the wound microenvironment were identified later. It was judiciously concluded that wounds with a pathologic inflammatory state and a high proteolytic microenvironment are a hostile battlefield for GFs and their receptors. It was also demonstrated that, in addition to being degraded, topical GF administration does not ensure adequate local diffusion to responsive cells in deeper layers [113]. These obstacles have contributed to GFs’ pharmaceutical applications fading in the wound healing armamentarium, and they explain why the FDA has not approved another GF-based medication for wound healing in more than 25 years. This is not to mention the limitations of recreating the full pathologic spectrum of chronic human wounds in laboratory animals. The present-day scientific community has reacted by unleashing a dense surge of novel and complex GF delivery and formulation technologies, whose cost-effectiveness and profitability are visibly uncertain. In parallel, the old ideas from the 1990s of administering GF combinations or administering GFs in a sequential manner have been reborn. These hypotheses will be destined to wither on the vine unless the following is identified: which is the optimal combination of GFs, the critical wound phase to apply them, the effective dose of each ingredient, and how does one ensure their chemical, physical, and biological integrity within the chronic wound milieu. The challenge ahead is big and appears unfounded when our real knowledge about most GFs in wound biology is still merely descriptive. The development of the intralesional infiltration of EGF in diabetic ulcers is founded on a series of sobering basic studies and validated by more than 20 years of clinical experience. This delivery route, aside from breaking diabetic wounds’ initial stagnancy and promoting the progressive growth of granulation tissue, enhances and sustains durable wound coverage with exceptionally low recurrence rates as compared to cutting-edge treatments. Studies have documented that EGF infiltration to diabetic wound bottoms, walls, and derma-epidermal junctions triggers a cascade of local fibroblast reactions, suggesting its molecular integrity, prolonged mean residence time, and, ultimately, its receptor trafficking and nuclear translocation, enhancing the transcription of critical genes. Furthermore, this delivery route, in addition to being safe in the short and long terms, has been shown to improve a series of biochemical parameters recurrently disturbed in diabetic patients. Therefore, this delivery method, aside from having assisted in preserving thousands of limbs, has vindicated the full spectrum of EGF intrinsic biological potency as a master driver of tissue rebuilding and rejuvenation—tagging it as the standalone polypeptide among the rest of the GFs present in the tissue repair kingdom.

## Figures and Tables

**Table 1:** Systemic effects of locally infiltrated EGF in patients with diabetic foot ulcers.

System	Systemic Effect
Redox balance	↓ Total oxidative capacity
	↓ MDA
	↓ AOPP
	↓ Total organoperoxides
	↓ Nitrite/nitrate ratio
	↑ Total antioxidant capacity
	↑ SH groups
Anti-inflammatory mechanism	↓ Erythrosedimentation
	↓ C reactive protein
	↓ IL-6
	↓ MIP1-α
	↓ sFAS
AGE pathway	↓ Pentosidine
	↑ sRAGE
Extracellular matrix	↓ MMP-9
	↓ TIMP-1

MDA: malondialdehyde; AOPP: advanced oxidation protein product; SH: sulfhydryl group; IL-6: interleukin-6; MIP1-α: macrophage inflammatory protein 1-alpha; sFAS: soluble FAS; AGE: advanced glycation end product; sRAGE: soluble receptor for AGE; MMP-9: matrix metalloprotease 9; TIMP-1: tissue inhibitor of MMP 1.

## Abbreviations

DFU	diabetic foot ulcer
EGF	epidermal growth factor
EGFR	epidermal growth factor receptor
ERK	extracellular-regulated kinase
ETF	EGFR-specific transcription factor
FDA	Food and Drug Administration
FGF	fibroblast growth factor
FOXO	forkhead box O
GFs	growth factors
HB-EGF	heparin-binding EGF-like growth factor
HMEC	human mammary epithelial cell
IGF	insulin-like growth factor
IL-6	interleukin-6
MAPK	mitogen-activated protein kinase
MIP1-α	macrophage inflammatory protein 1-alpha
mTORC	mammalian target of rapamycin complex
NGF	nerve growth factor
PDGF	platelet-derived growth factor
PDK1	pyruvate kinase
PI3K	phosphatidylinositol 3-kinase
PIP3	phosphatidylinositol triphosphate
PLCγ1	phospholipase C gamma 1
Rb	retinoblastoma
sFAS	soluble FAS
TGF-α	transforming growth factor alpha
TGF-β	transforming growth factor beta
VAC	vacuum-assisted closure
VEGF	vascular endothelial growth factor
